# Supplementary material for: Echinococcus granulosus antigen B acts as an LPS-scavenging lipoprotein in vitro, preventing TLR4-mediated activation of dendritic cells
Source: Infect Immun. 2025 Dec 16;94(1):e00361-25. doi: 10.1128/iai.00361-25 (PMC12798045; doi:10.1128/iai.00361-25)
Supplement: Supplemental figures — Fig. S1 to S7. [file iai.00361-25-s0001.pdf]

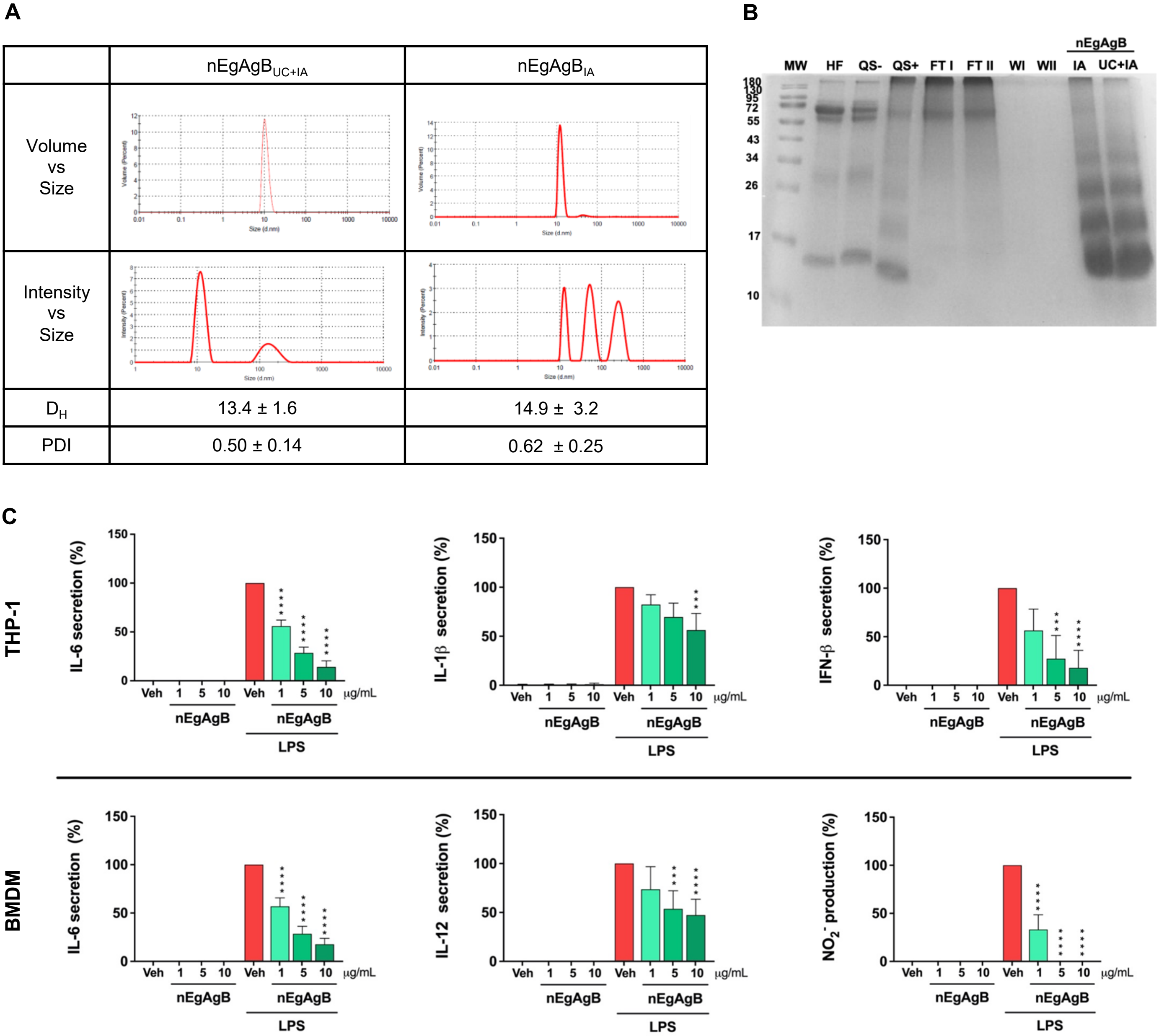

Supplementary Figure 1. nEgAgB purification from HF by ion exchange chromatography followed by immunoaffinity chromatography (IA) resulted in a lipoprotein particle (nEgAgB$_{IA}$) with properties comparable to the lipoprotein obtained using ultracentrifugation (UC) combined with IA (nEgAgB$_{UC+IA}$). These similarities were confirmed by analysis of the size protein composition and immunomodulatory properties. Panel (A) shows representative dynamic light scattering (DLS) data for nEgAgB$_{IA}$ and nEgAgB$_{UC+IA}$ (weighted by intensity and volume), including data corresponding to the hydrodynamic diameter (D_H, nm) and polydispersity index (PDI) expressed as the mean and standard deviation of two independent preparations. Panel (B) corresponds to SDS-PAGE electrophoresis (15% polyacrylamide, 6 mM DTT, Colloidal Coomassie Blue staining) of fractions from the nEgAgB$_{IA}$ purification protocol: bound (QS+) and unbound (QS-) fractions to Q-Sepharose, and bound (nEgAgB$_{IA}$) and unbound fractions (FT, flow through I and II; and W, washes I and II with PBS$_{EBAb}$ or glicine pH 5, respectively) to the anti-EgAgB clone1-Sepharose (14). For comparison, HF and nEgAgB$_{UC+IA}$were analysed in parallel. The nEgAgB$_{IA}$ showed a similar pattern compared to nEgAgB$_{UC+IA}$, with regularly spaced bands of ~8, 16, 24 and 32 kDa corresponding to the monomer and its oligomeric forms. Panel (C) illustrates data from the analysis of immunomodulatory activity of nEgAgB$_{IA}$ on the LPS-induced activation in PMA-differentiated THP-1 macrophages and murine bone marrow-derived macrophages (BMDM), following the methodology described in our previous work (14). BMDM were prepared using the procedure approved by CHEA (protocol N° 538, Exp. No. 101900-000999-17, UdelaR, Uruguay). nEgAgB$_{IA}$ reproduced the inhibition of cytokine secretion (IL-1β, IL-6, IL-12p40, IFN-β) and nitric oxide (NO·) generation observed for nEgAgB$_{UC+IA}$. The data is presented as the mean and standard deviation of values normalised to LPS/Veh condition (set as 100%) and correspond to three independent experiments with analytical duplicates. Statistical analysis was performed using raw data and significant differences compared to LPS/Veh are indicated with * (two-way ANOVA and Tukey's test, ** $p < 0.01$, *** $p < 0.001$, **** $p < 0.0001$).

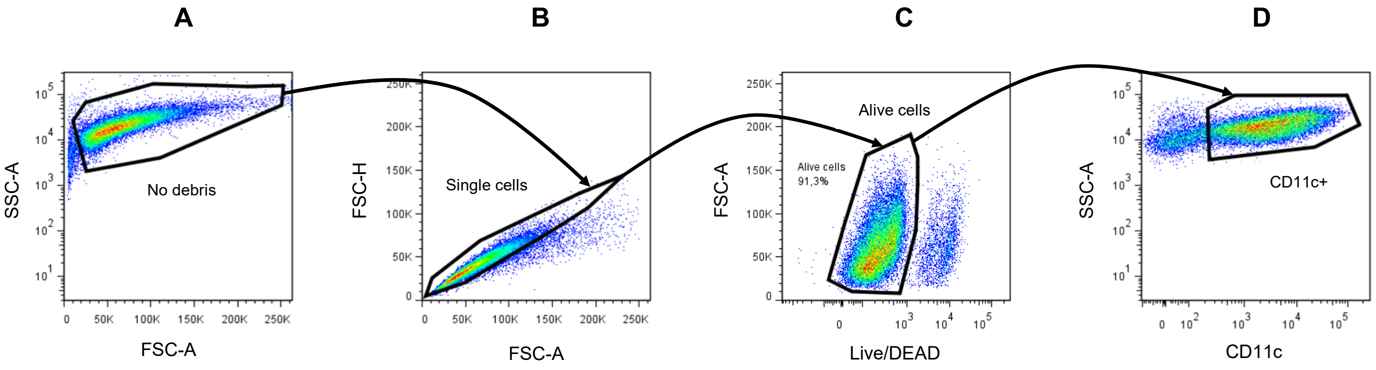

Supplementary Figure 2. Gating strategy for dendritic cell definition. Following the bone marrow dendritic cell generation protocol, non-adherent cells were harvested at day 10 and stained with Live/DEAD-Green and anti-CD11c-PECy7, among other antibodies (depending on the experimental activity). The gating strategy to define dendritic cells consisted on selecting: A) cells (excluding debris), B) singlets (excluding doublets), C) alive cells (excluding Live/DEAD^{+}) and D) dendritic cells (Live/DEAD^{-}CD11c^{+} events).

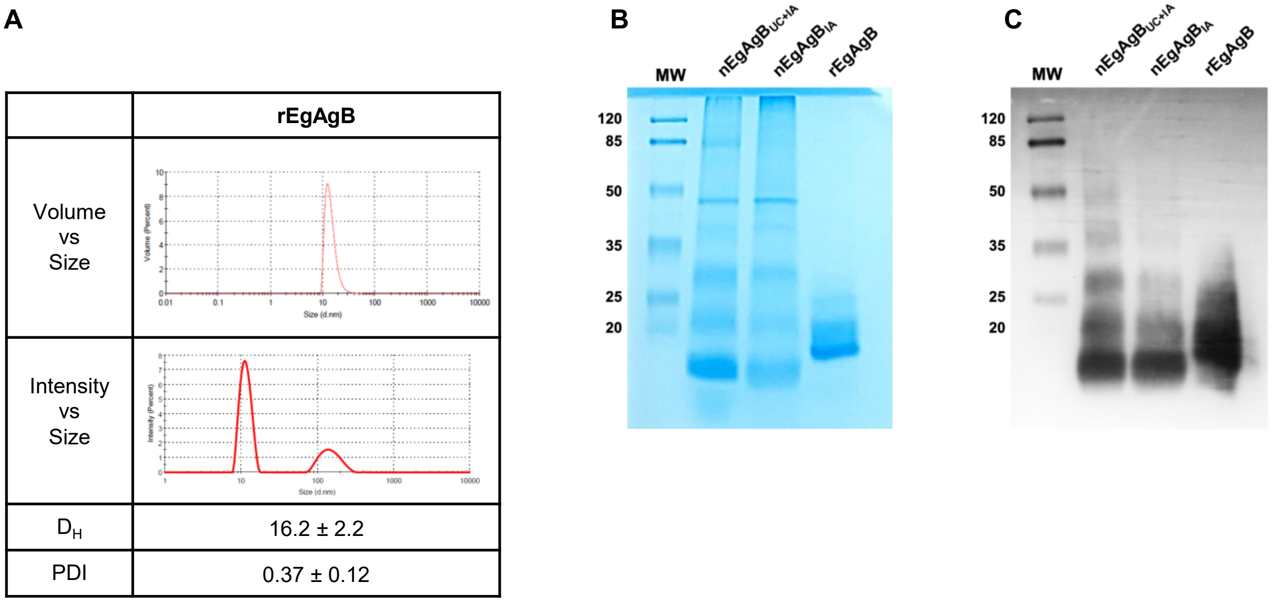

Supplementary Figure 3. Molecular size of rEgAgB and protein profile of nEgAgB and rEgAgB. A) Analysis of rEgAgB by dynamic light scattering (DLS). Representative graphs of the hydrodynamic diameter (D_H) weighted by volume or intensity are shown; the D_H and polydispersity index (PDI) are expressed as the mean and standard deviation of two independent preparations analyzed in triplicates. B) Analysis by SDS-PAGE electrophoresis (15% polyacrylamide, 6 mM DTT, Colloidal Coomassie Blue staining) of nEgAgB purified by the three-step fractionation protocol based on an ion exchange chromatography on Q-Sepharose, followed by sequential ultracentrifugation and a final immunoaffinity chromatography ($nEgAgB_{UC+IA}$) or without the ultracentrifugation step ($nEgAgB_{IA}$), and rEgAgB. C) Analysis of $nEgAgB_{UC+IA}$, $nEgAgB_{IA}$ and rEgAgB by Western blot using the mAb EB7 (39).

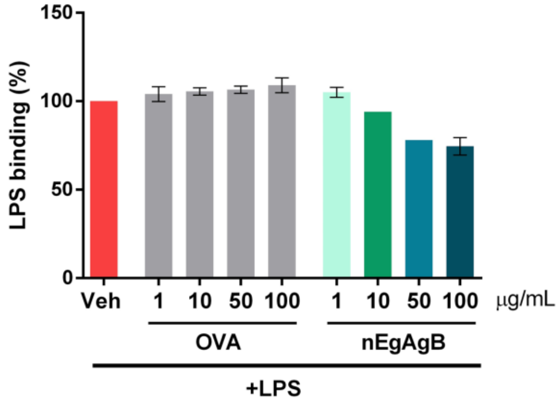

Supplementary Figure 4. nEgAgB competes with LPS for binding to BMDCs, as indicated by reduced fluorescence from LPS-Alexa Fluor 488 after preincubation with nEgAgB. BMDC ($0.2x10^6$) were pre-incubated with nEgAgB, OVA (both at concentrations of 1-100 µg/mL), or PBS_{EBAb} (Veh), and subsequently with LPS-Alexa Fluor 488. After 30 min, cells were washed and the fluorescence was measured using a FACSCanto II cytometer (BD Biosciences). LPS binding is plotted as the median and range of data normalised to the LPS/Veh condition (set as 100%). The data correspond to one representative experiment performed with analytical duplicates.

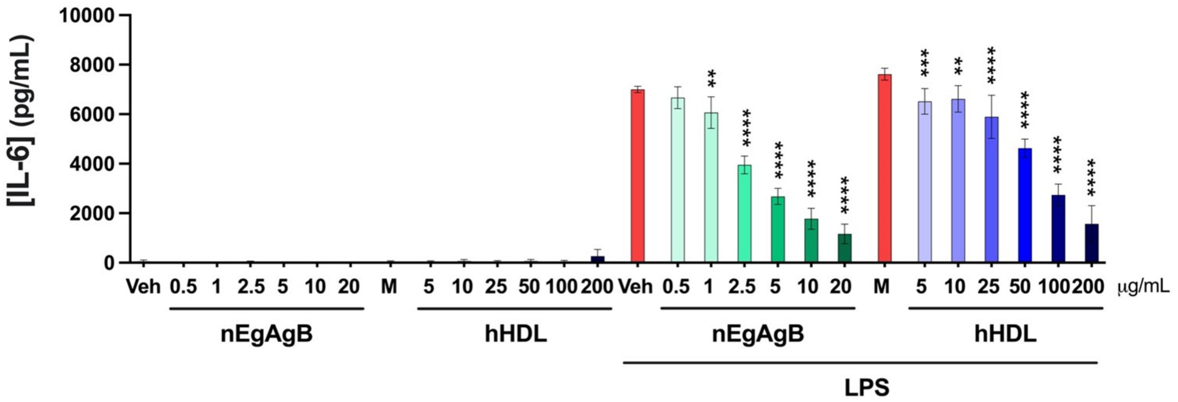

Supplementary Figure 5. nEgAgB inhibited LPS-induced activation on THP-1 macrophages, as HDL does. THP-1 macrophages ($0.2x10^6$) were stimulated with $nEgAgB_{IA}$ (0.5-20 µg/mL), HDL (5-200 µg/mL) in absence or presence of LPS (10 ng/mL), using PBS_{EBAb} as a vehicle control (Veh). After 24 hs, the culture supernatants were collected to determine IL-6 secretion by ELISA. The data are plotted as the mean ± SD and correspond to three independent experiments with analytical duplicates. Significant differences compared to Veh+LPS are indicated with * (two-way ANOVA and Tukey's test, ** $p < 0.01$, *** $p < 0.001$, **** $p < 0.0001$).

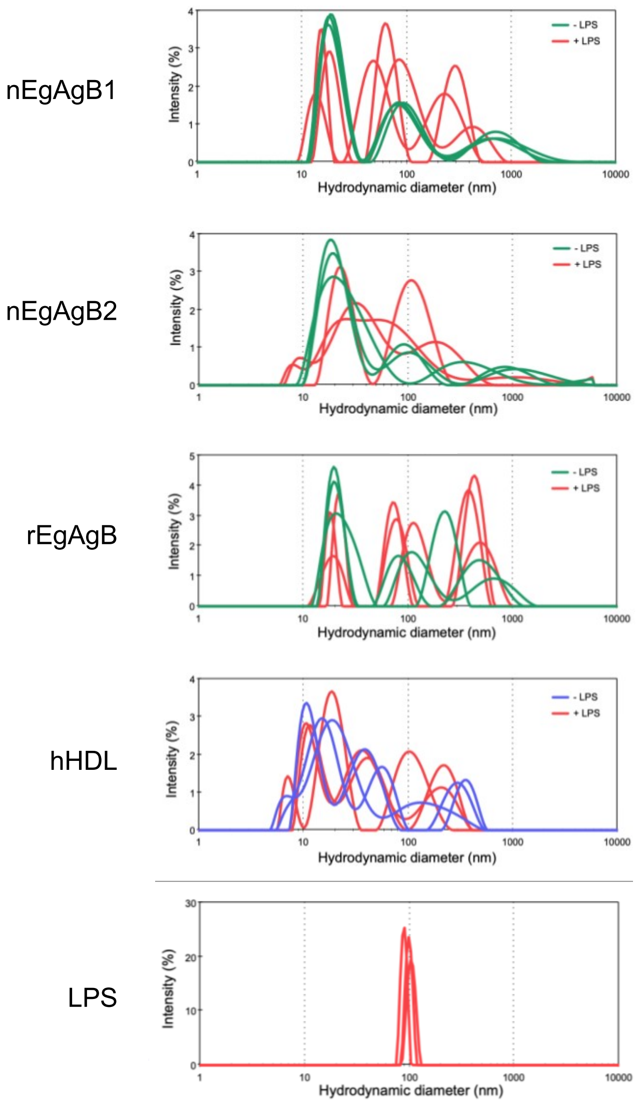

Supplementary Figure 6. Analysis of EgAgB-LPS interaction by DLS. nEgAgB/rEgAgB, hHDL (1 mg/mL) or its vehicle (PBS_{EBAb}) were analysed by DLS in the presence of LPS (1 mg/mL) or its vehicle (PBS). Two preparations of nEgAgB (1 and 2) were used. The size distribution weighted by intensity of each population (3 consecutive measurements) is plotted in the absence (green and blue lines for EgAgB and hHDL, respectively) or presence of LPS (red lines). LPS modified the size distribution of EgAgB in solution while this alteration was not as noticeable for hHDL.

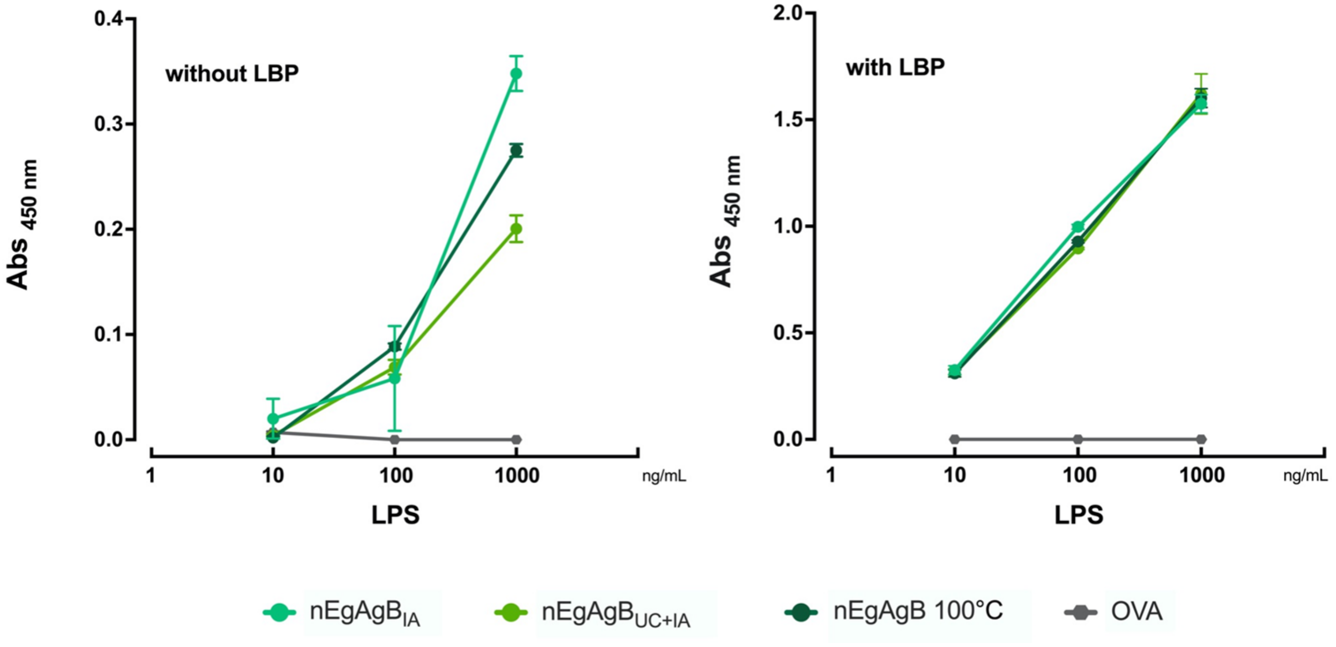

Supplementary Figure 7. LPS-binding activity was thermostable and comparable between nEgAgB$_{IA}$ and EgAgB$_{UC+IA}$. nEgAgB$_{UC+IA}$, nEgAgB$_{IA}$ with or without a heat-treatment (100°C for 10 min) or OVA (10 µg/mL, as an irrelevant protein control) were immobilised in ELISA microplates by overnight incubation at 4°C. As a control, wells were incubated with PBS. After blocking with PBS-BSA 1%, LPS-biotin (1, 10, 100 and 1000 ng/mL) was added in the absence or presence of hLBP (0.1 µg/mL). The interaction of LPS-biotin with the immobilised macromolecules was revealed by streptavidin-HRP addition followed by HRP activity determination. The absorbance (450 nm) is plotted as the mean ± SD of data and graphs are representative of two independent experiments with analytical duplicates.
