# Supplementary material for: Echinococcus granulosus antigen B acts as an LPS-scavenging lipoprotein in vitro, preventing TLR4-mediated activation of dendritic cells
Source: Infect Immun. 2025 Dec 16;94(1):e00361-25. doi: 10.1128/iai.00361-25 (PMC12798045; doi:10.1128/iai.00361-25)
Supplement: Supplemental tables — Tables S1 and S2. [file iai.00361-25-s0002.docx]

**Supplementary Tables**

**Supplementary Table 1.** Fluorescent conjugates employed in the analyses by flow cytometry

| **Fluorochrome** | **Antigen** | **Clone** | **Final dilution** | **Brand** | **Catalogue** |
| --- | --- | --- | --- | --- | --- |
| FITC | CD14 | Sa14-2 | 1/200 | Biolegend | 123308 |
| PE | CD86 | GL-1 | 1/200 | Biolgened | 105008 |
| PE | CD284/MD2 complex | MTS510 | 1/200 | Biolegend | 117606 |
| PECy7 | CD11c | N418 | 1/200 | Biolegend | 117318 |
| APC | CD40 | 3/23 | 1/200 | Biolegend | 124612 |
| APC | CD284 | SA15-21 | 1/200 | Biolegend | 145406 |

**Supplementary Table 2. Molecular docking of EgAgB8/1 and LPS interaction**

Molecular docking analyses of EgAgB8/1 (accession number U6JQF4) interaction with R2 core and R3 core, lipid A and antigen O regions of LPS using HADDOCK. All LPS regions tested were capable of interacting with EgAgB8/1 forming a substantial number of hydrogen bonds that contributed to the stabilization of the complexes. Among the most recurrent interacting residues were Arg66, Arg70, Glu59, and Phe62, which established multiple hydrogen bonds and salt bridges with the lipid A and R3 core. The O-antigen and R2 core domains exhibited comparatively fewer interactions, with Glu59 and Gln63 as the primary contributors. Hydrophobic contacts with Phe42, Leu47, and Val51 further stabilized the lipid A-EgAgB8/1 complex.

**Core R2**

| **Hydrophobic Interactions** | | | | |
| --- | --- | --- | --- | --- |
| **Residue** | **AA** | **Distance (Å)** | **Ligand Atom** | **Protein Atom** |
| 63 | GLN | 3.63 | 910 | 527 |

| **Hydrogen Bonds** | | | | | | |
| --- | --- | --- | --- | --- | --- | --- |
| **Residue** | **AA** | **Distance H-A (Å)** | **Distance D-A (Å)** | **Donor Angle** | **Donor Atom** | **Acceptor Atom** |
| 8 | ALA | 2.37 | 3.32 | 163.93 | 1[N3] | 850 [O3] |
| 52 | VAL | 3.28 | 3.63 | 102.80 | 870 [O3] | 423 [O2] |
| 59 | GLU | 3.04 | 3.57 | 114.31 | 857 [O3] | 490 [O2] |
| 62 | PHE | 2.97 | 3.63 | 124.89 | 908 [O3] | 522 [O2] |
| 66 | ARG | 3.19 | 3.90 | 126.74 | 562 [Ng+] | 898 [O3] |
| 67 | LYS | 3.16 | 3.66 | 111.02 | 577 [N3+] | 927 [O3] |
| 70 | ARG | 2.12 | 2.94 | 134.05 | 617 [Ng+] | 923 [O3] |

| **Salt Bridges** | | | | |
| --- | --- | --- | --- | --- |
| **Residue** | **AA** | **Distance (Å)** | **Ligand Group** | **Ligand Atoms** |
| 66 | ARG | 3.99 | Carboxylate | 906 - 907 |

**Core R3**

| **Hydrogen Bonds** | | | | | | |
| --- | --- | --- | --- | --- | --- | --- |
| **Residue** | **AA** | **Distance H-A (Å)** | **Distance D-A (Å)** | **Donor Angle** | **Donor Atom** | **Acceptor Atom** |
| 8 | ALA | 3.54 | 3.99 | 110.10 | 1 [N3] | 770 [O3] |
| 11 | LEU | 3.31 | 3.99 | 127.80 | 22 [Nam] | 776 [O3] |
| 13 | SER | 2.76 | 3.48 | 132.13 | 43 [O3] | 857 [O3] |
| 50 | LYS | 2.37 | 3.25 | 146.00 | 806 [O3] | 407 [O2] |
| 51 | VAL | 2.02 | 2.53 | 109.56 | 809 [O3] | 415 [O2] |
| 54 | LEU | 2.39 | 3.37 | 165.39 | 812 [O3] | 441 [O2] |
| 62 | PHE | 2.13 | 2.50 | 102.47 | 903[O. co2] | 522 [O2] |
| 62 | PHE | 2.14 | 2.02 | 145.94 | 905 [O3] | 522 [O2] |
| 66 | ARG | 2.52 | 3.16 | 123.02 | 559 [Ng+] | 924 [O3] |
| 66 | ARG | 2.50 | 2.90 | 102.17 | 565 [Ng+] | 927 [O3] |
| 66 | ARG | 3.12 | 3.46 | 102.05 | 553 [Nam] | 903[O.co2] |
| 70 | ARG | 2.90 | 3.79 | 151.82 | 611 [Ng+] | 924 [O3] |

**Lipid A**

| **Hydrophobic Interactions** | | | | |
| --- | --- | --- | --- | --- |
| **Residue** | **AA** | **Distance (Å)** | **Ligand Atom** | **Protein Atom** |
| 41 | PHE | 2.95 | 847 | 309 |
| 42 | PHE | 3.18 | 842 | 321 |
| 42 | PHE | 3.50 | 840 | 322 |
| 42 | PHE | 3.98 | 839 | 320 |
| 47 | LEU | 3.06 | 847 | 375 |
| 51 | VAL | 3.79 | 844 | 413 |
| 51 | VAL | 3.27 | 843 | 411 |
| 52 | VAL | 3.17 | 839 | 421 |
| 55 | LEU | 3.42 | 838 | 447 |
| 56 | LYS | 3.21 | 881 | 456 |
| 57 | GLU | 3.87 | 887 | 468 |
| 63 | GLN | 3.16 | 777 | 527 |
| 70 | ARG | 3.37 | 804 | 608 |
| 71 | MET | 3.66 | 804 | 625 |

| **Hydrogen Bonds** | | | | | | |
| --- | --- | --- | --- | --- | --- | --- |
| **Residue** | **AA** | **Distance H-A (Å)** | **Distance D-A (Å)** | **Donor Angle** | **Donor Atom** | **Acceptor Atom** |
| 8 | ALA | 2.57 | 3.48 | 154.86 | 1 [N3] | 836 [O2] |
| 63 | GLN | 3.01 | 3.36 | 102.31 | 786 [Nam} | 529 [O2] |
| 63 | GLN | 3.15 | 3.82 | 125.65 | 775 [O3] | 534 [O2] |
| 70 | ARG | 2.56 | 3.06 | 109.12 | 617 [Ng+] | 775 [O3] |

**Antigen O**

| **Hydrophobic Interactions** | | | | |
| --- | --- | --- | --- | --- |
| **Residue** | **AA** | **Distance (Å)** | **Ligand Atom** | **Protein Atom** |
| 55 | LEU | 3.39 | 779 | 445 |
| 59 | GLU | 3.48 | 828 | 486 |

| **Hydrogen Bonds** | | | | | | |
| --- | --- | --- | --- | --- | --- | --- |
| **Residue** | **AA** | **Distance H-A (Å)** | **Distance D-A (Å)** | **Donor Angle** | **Donor Atom** | **Acceptor Atom** |
| 8 | ALA | 3.01 | 3.55 | 116.29 | 1 [N3] | 809 [O3] |
| 42 | PHE | 3.26 | 3.92 | 126.09 | 314 [Nam] | 764 [O3] |
| 56 | LYS | 3.17 | 3.49 | 118.93 | 458 [N3+] | 792 [O2] |
| 58 | LEU | 2.93 | 3.54 | 121.14 | 474 [Nam] | 827 [O2] |
| 58 | LEU | 2.27 | 3.04 | 131.91 | 820 [O3] | 482 [O2] |
| 59 | GLU | 2.38 | 3.27 | 156.89 | 490 [O3] | 831 [O3] |
| 59 | GLU | 2.45 | 3.27 | 138.18 | 831 [O3] | 490 [O3] |
| 59 | GLU | 3.54 | 3.85 | 101.09 | 483 [Nam] | 827 [O2] |
| 59 | GLU | 3.13 | 3.84 | 129.79 | 799 [Nam] | 489 [O2] |
| 59 | GLU | 2.67 | 3.53 | 145.30 | 796 [O3] | 489 [O2] |
| 63 | GLN | 2.43 | 3.18 | 132.67 | 530 [Nam] | 803 [O2] |
